# Diversity and Global Distribution of Viruses of the Western Honey Bee, *Apis mellifera*

**DOI:** 10.3390/insects11040239

**Published:** 2020-04-10

**Authors:** Alexis Beaurepaire, Niels Piot, Vincent Doublet, Karina Antunez, Ewan Campbell, Panuwan Chantawannakul, Nor Chejanovsky, Anna Gajda, Matthew Heerman, Delphine Panziera, Guy Smagghe, Orlando Yañez, Joachim R. de Miranda, Anne Dalmon

**Affiliations:** 1 Institute of Bee Health, Vetsuisse Faculty, University of Bern, 3003 Bern, Switzerland; orlando.yanez@vetsuisse.unibe.ch; 2 Agroscope, Swiss Bee Research Center, 3003 Bern, Switzerland; 3 UR Abeilles et Environnement, INRAE, 84914 Avignon, France; anne.dalmon@inrae.fr; 4 Laboratory of Agrozoology, Department of Plants and Crops, Faculty of Bioscience Engineering, Ghent University, 9000 Ghent, Belgium; Niels.Piot@UGent.be (N.P.); Guy.Smagghe@UGent.be (G.S.); 5 Institute of Evolutionary Ecology and Conservation Genomics, University of Ulm, 86069 Ulm, Germany; vincent.doublet@uni-ulm.de; 6 Department of Microbiology, Instituto de Investigaciones Biológicas Clemente Estable, Montevideo 11600, Uruguay; kantunez03@gmail.com; 7 Centre for Genome Enabled Biology and Medicine, University of Aberdeen, Aberdeen AB24 3FX, UK; e.m.campbell@abdn.ac.uk; 8 Environmental Science Research Center (ESRC), Faculty of Science, Chiang Mai University, Chiang Mai 50200, Thailand; panuwan@gmail.com; 9 Bee Protection Laboratory (BeeP), Department of Biology, Faculty of Science, Chiang Mai University, Chiang Mai 50200, Thailand; 10 Entomology Department, Institute of Plant Protection, The Volcani Center, Rishon Lezion, Tel Aviv 5025001, Israel; ninar@volcani.agri.gov.il; 11 Laboratory of Bee Diseases, Institute of Veterinary Medicine, Warsaw University of Life Sciences, 02-787 Warsaw, Poland; anna_gajda@sggw.pl; 12 Bee Research Lab, USDA-ARS, Beltsville, MD 20705, USA; matthew.heerman@usda.gov; 13 Institute of Biology, Martin-Luther-University Halle-Wittenberg, 06120 Halle (Saale), Germany; delphine.panziera@gmx.com; 14 German Centre for Integrative Biodiversity Research (iDiv) Halle-Jena-Leipzig, 04103 Leipzig, Germany; 15 Department of Ecology, Swedish University of Agricultural Sciences, 750-07 Uppsala, Sweden; joachim.de.miranda@slu.se

**Keywords:** epidemiology, emerging infectious diseases, pathogens, invasive species, social insects, viruses, honey bee health

## Abstract

In the past centuries, viruses have benefited from globalization to spread across the globe, infecting new host species and populations. A growing number of viruses have been documented in the western honey bee, *Apis mellifera*. Several of these contribute significantly to honey bee colony losses. This review synthetizes the knowledge of the diversity and distribution of honey-bee-infecting viruses, including recent data from high-throughput sequencing (HTS). After presenting the diversity of viruses and their corresponding symptoms, we surveyed the scientific literature for the prevalence of these pathogens across the globe. The geographical distribution shows that the most prevalent viruses (deformed wing virus, sacbrood virus, black queen cell virus and acute paralysis complex) are also the most widely distributed. We discuss the ecological drivers that influence the distribution of these pathogens in worldwide honey bee populations. Besides the natural transmission routes and the resulting temporal dynamics, global trade contributes to their dissemination. As recent evidence shows that these viruses are often multihost pathogens, their spread is a risk for both the beekeeping industry and the pollination services provided by managed and wild pollinators.

## 1. Introduction

There is a general consensus that the industrial revolution and increase in global trade have greatly altered the geographical distribution of species [1,2,3]. As a result, outbreaks of emerging infectious diseases (EIDs) have become more frequent, and these are currently considered to be one of the most critical threats to wildlife, public health and food security [4,5,6]. Moreover, the threats caused by EIDs are compounded by an increase in the re-emergence of previously known diseases, caused by a variety of factors such as the evolution of antibiotic resistant pathogens [7] and the increase in the global transport of humans, plants and animals and the products derived from them [8]. In a context of global change, understanding the distribution and spread of diseases is essential to designing appropriate control and containment strategies [9,10].

Viruses stand among the most diverse and widespread agents causing EIDs [11,12]. Due to their high evolutionary capacity and exponential replication rates, these pathogens can readily adapt to new hosts and cause large-scale epidemics [13,14,15,16]. In parallel to the increasing risks posed by these pathogens, the continual advances in molecular biology have greatly increased our capacity to identify new microorganisms [17,18,19], leading to an explosion of new virus discoveries recently that greatly challenge our understanding of the complexity of life [20,21] and the possible roles and origins of viruses [22,23].

Viruses have been linked to a number of honey bee diseases [24] and can act synergistically with other biotic and abiotic stressors, which can lead to the collapse of host colonies [25,26,27]. Additionally, there is growing evidence that viruses infecting honey bees can affect a wide range of other insect species [28,29,30] and therefore threaten our ecosystems more profoundly than previously thought. In the last decades, numerous studies have been conducted to assess the prevalence of viruses in honey bee colonies throughout the world, mostly using molecular diagnostic tools. The aim of this review is to provide a comprehensive survey of the diversity and distribution of viruses known to infect and replicate in *Apis mellifera*. In this review, we first described the diversity of viruses infecting the western honey bee and their associated symptoms. In the second part we conducted an extensive literature survey of the geographical distribution of the major viruses infecting *A. mellifera* and reviewed their temporal population dynamics. In the third part, we discussed the multiple factors that influence the spatiotemporal distribution of viruses. Finally, we scanned the potential knowledge gaps of the field and identified new perspectives to tackle the future challenges associated with the viruses of *A. mellifera*.

## 2. Diversity of Viruses of the Western Honey Bee

### 2.1. What Are the Viruses of the Western Honey Bee?

Viruses are obligate intracellular infectious agents that rely on their host machinery for multiplication (i.e., transcription, translation and replication) (Box 1). By doing so, they can cause significant damage to their hosts and express a variety of symptoms. In honey bees, viral infections have been reported to affect diverse traits, such as morphology, physiology and behavior; in severe cases, viruses may lead to increased mortality at the individual as well as colony levels. For example, wings deformities and shortened abdomens are characteristic symptoms of deformed wing virus (DWV) infection [31]. However, some bee viruses have been recently discovered that show no apparent symptoms, or only mild symptoms, in infected honey bees [32,33,34]. For the purposes of this review, we define “viruses of the honey bee” as viruses that replicate in honey bees, in contrast to the term “honey bee virus” that implies a single-host pathogen, rather than the multi-host pathogens that these viruses are known to be (Box 1).

Like in most other organisms, viruses with RNA genomes predominate in honey bees. These viruses typically consist of small icosahedral particles (17, 30 or 35 nm) that contain a positive-sense, single-stranded RNA genome (ranging from 1.1–10 kbp) [24,35,36]. After attaching to and penetrating into their host cells, these viruses generally replicate by injecting RNA directly into the cytoplasm. The host machinery (e.g., ribosomes) will then transcribe and generate new viral proteins, that will be assembled as a viral particle, released from the infected cell and will start infecting new cells. More recently, RNA viruses with a negative-sense, single-stranded genome, which are generally more difficult to propagate *in vivo*, have also been identified in *A. mellifera* [32,33,34]. Additionally, a few viruses with a DNA genome have been identified in honey bees. These DNA viruses differ from RNA viruses in the way they replicate. Once within their host cells, DNA viruses are transported to the host nucleus to be transcribed and translated. Although this group of viruses has been known to infect *A. mellifera* for decades [37], studies involving DNA viruses remain much rarer than those on RNA viruses.

Box 1 **Terms and definitions.**

**Host:** In this review, we define the *host* of a virus as the organism in which the pathogen actively replicates.

**Virus detection:***Virus detection* is the establishment of the presence of a particular virus in an organism, using a variety of technologies. The mere detection of a virus in the organism does not necessarily indicate that the pathogen is using the host to replicate.

**Prevalence:** The *prevalence* of a virus is the proportion of a specified target group (e.g., populations) that is infected by that virus. In honey bees, the prevalence of viruses can be described at a wide range of scales, from organs [38] to species [39] and landscape levels.

**Replication:** Virus *replication* refers to the multiplication of the virus within the host’s cells. Viruses rely on the host’s cellular machinery to replicate. Eventually, the replication of viruses may impair host fitness or result in the host’s death.

**Symptoms:***Symptoms* are the physical or behavioral changes caused by the host’s adverse reaction to the presence and replication of a virus. The most striking virus symptom in honey bees are the wing malformations caused by DWV.

### 2.2. Diversity of Viruses of the Honey Bee

Honey bee virology was relatively simple prior to the advent of cheap, fast, high-throughput sequencing (HTS) technologies. The only viruses that one knew or cared about were those that revealed themselves through symptoms: either physical (DWV, CWV, AmFV, AIV—see Table 1), developmental (SBV, BQCV), behavioral (CBPV, ABPV, SBPV) or demographic (BVX, BVY) (Table 1). The main task was to confirm Koch’s postulates [40] or its modern equivalents [41,42] linking a virus to the symptoms, which simultaneously confirmed the honey bee’s host status for the virus. The existence of other viruses was suspected and even sometimes revealed as unintended by-products of propagation [24,36], as was the possibility of more extensive host ranges for these viruses [28,29,30]. However, the technological limitations of the day meant that other, asymptomatic viruses remained largely unknown.

HTS technologies have effectively turned this entire paradigm upside-down by uncovering, through sequencing and bioinformatic analyses, a staggering diversity of previously unknown viruses that vastly outstrips the viruses known from diseases [23]. These discoveries have shown that the overwhelming majority of viruses are asymptomatic, and that only a miniscule proportion cause disease [22]. This avalanche of virus discoveries covers all organisms and environments, surpassing the capacity of the existing taxonomic structures to catalogue and organize this data, while the discoveries themselves add resolution and power to the bioinformatics tools for further discoveries, in new or old datasets. For bees, this process started in earnest with the mass screening for possible virological links to colony collapse disorder (CCD) [43], with each subsequent effort adding to the virus diversity in bees or its ectoparasite *Varroa destructor* [32,33,34,44,45,46,47,48], culminating in the list in Table 1. However, this is almost certainly a vast underestimate of the true total virus diversity in bees, judging by the rate of new discoveries [49], not only of virus species and genera [50] but also of families and orders [23]. Additionally, methodological biases exist in the discovery process towards RNA viruses (preponderance of transcriptome studies in HTS), especially those with poly-A tail (e.g., Picornavirales), and a bioinformatic screening based largely on conserved replication protein domains, thereby excluding subviral entities lacking a replicase (viroids, satellite viruses), viruses replicating by other means and other non-host, replicating, bioactive nucleic acid sequences.

The extent of this virological biodiversity by itself poses questions about its origins and functional significance, either as former/future pathogens or molecular symbionts [22]. Impressive as it is, this accumulation of honey-bee-derived virus genome sequences needs to be completed by molecular, biological and epidemiological character studies in order to understand their possible functional and ecological roles [22,29,30] and thus their current relevance and possible future significance. For the importance of adding biological flesh and personality to these sequences, one has to look no further than DWV, which was exactly one of these thousands of unassuming, asymptomatic viruses until it was weaponized by *V. destructor* into becoming the scourge that it is today [51].

### 2.3. Symptoms of Viral Infections of the Honey Bee

The pathogenicity of viruses is the consequence of their replication within the cells of diverse organs of honey bees. Some of the viruses have tropism towards many organs, while other viruses are restricted to certain specific organs (Table 2). Thus, if symptoms appear in an individual bee, they are a direct outcome from the virus interrupting the function of one or more organs/systems in the bee’s body. Clear symptoms usually only appear at very high virus titers, and many persistent or asymptomatic infections that may cause long-term damage to the colony can remain undetected [52]. On the other hand, symptoms of viral infections can be obscured by those coming from other diseases, or environmental stressors (e.g., chemical poisoning, honeydew poisoning and the “black robber” syndrome in CBPV infection, see Table 2).

Many bee viruses do not produce clear physical or behavioral symptoms in honey bees, while for others symptoms may exist but have not yet been identified. The honey bee viral diseases that have been most extensively studied usually also possess one or more unique symptoms that enable a simple and reliable diagnosis for beekeepers. Virus pathology, symptom development and virulence depends on several factors, the most important of which are the amount of virus produced, where it is produced (tissue/organ localization) and its transmission route [53,54]. The complexity of studying virus virulence and symptom development at individual and colony level has been revealed in particular for certain well-studied viruses such as the different strains of deformed wing virus (Box 2). The typical symptoms of this pathogen, wing deformities, are closely linked to transmission by *V. destructor* or injection [55,56,57]. By contrast, it appears that infection through vertical, venereal or oral transmission is effectively symptomless, even if the virus titers in the affected organs can be as high as those achieved by Varroa-mediated transmission [41,49,50]. Finally, the relationship between titers and symptoms is not always straightforward, and covert infections may result in more cryptic symptoms, such as changes in behavioral maturation that may be more difficult to discern [58,59,60].

Box 2 **Diversity and pathogenicity of deformed wing virus.**

The existence of major strains for any one virus species is of great significance for virulence evolution, quasispecies dynamics and virus adaptation [62]. As a consequence of the emergence of *V. destructor* as a novel, exotic mechanical, and sometimes also biological, vector of viruses in *A. mellifera*, different strains of deformed wing virus (DWV) have adapted to this new transmission route [63], leading to the emergence of new threats to honey bee health. Disentangling the individual effects of the different strains of this virus on honey bee health is complex, especially for colony-level effects. For instance, DWV-B (originally named *Varroa destructor* virus-1 or VDV-1) is an emerging strain of DWV with elevated virulence in continental Europe [64]. In studies conducted in Germany, this pathogen appeared to be more virulent than the other major DWV variant, DWV-A, at the individual bee level in adults workers [64,65] but not in pupae [66]. However, although DWV-B has recently been detected in the USA, its impact on honey bee health there is unclear [67], since DWV-A has been associated with higher overwintering colony losses across the country [68]. Moreover, these two DWV strains differ markedly in their interaction with *V. destructor*. Notably, DWV-B is capable of replicating in the mite [69,70], while DWV-A is not [71,72]. The higher virulence of DWV-B when transmitted by the mite to pupae [65] makes the brood more susceptible to detection by adult bees and may therefore encourage its removal from the colony by social immunity, similar to other highly virulent Varroa-transmitted viruses [73]. However, DWV-B also impairs adult bee cognitive function to a greater extent than DWV-A [65], potentially compromising social hygiene efficiency in general, which may benefit DWV-B to a greater extent than DWV-A [74]. The two strains may thus each be favored in different aspects of the Varroa–virus–bee relationship, which may determine which strain persists at colony level under different circumstances [64,68,75,76]. Additionally, the spread of *V. destructor* and the global trade in bees and bee products has led to the co-occurrence of different major virus strains that did not initially exist in sympatry. This has resulted in the appearance of many natural recombinant strains between DWV-A and B, representing the majority of the DWV variants in colonies from the UK and France [77,78,79]. Through recombination, the most potent features of either strain can be linked in a single genome, thus abolishing the need for cooperation and self-regulation between strains through the quasispecies social structure [62]. For this reason, recombinant viruses are often more virulent than either of the parental genomes [68,76,77,78,79,80]. Finally, the discovery of a third major DWV variant (DWV-C) [81], less common and possibly more virulent than either DWV-A or DWV-B in honey bees [68,82], and the confirmation of Egypt bee virus [36] as a fourth major DWV variant (de Miranda, unpublished) adds a further level complexity to the possible interactions between these major DWV strains and their recombinants.

Many viruses are present in seemingly healthy colonies as asymptomatic or unapparent infections. In most cases some promoting factor, like the abovementioned varroosis (Box 2) or environmental stressors such as confinement, starvation, chemical residues, cold, humidity and other pathogens (e.g., *Nosema* spp.) are necessary to turn those into a symptomatic and overt infection [52,83].

## 3. Distribution of Viruses of the Western Honey Bee

### 3.1. Global Dissemination of Viruses of the Honey Bee

Honey bee viruses can be transmitted horizontally, vertically or vectored by parasites, such as the mite *V. destructor*. In addition, viruses can be dispersed anthropogenically, along nodes of a network of existing managed colonies. As the latter has the greater consequences, and because this review concerns the dissemination of viruses on a global scale, we will focus here on human activities that drive the dispersal of bee viruses.

The intervention of humans in the life of honey bees, through honey hunting, beekeeping and pollination services, has greatly expanded the range across which bee diseases can be spread. Humans have collected and consumed honey since prehistoric times [100] and have reared honey bees for honey production since the first emergence of large, urbanized civilizations in Europe and Asia. Even during the earliest days of managed beekeeping, there was a wide-ranging exchange of beekeeping products, knowledge, techniques and even favored bee strains and races. The improvement in beekeeping management, technology, bee breeding, transport and infrastructure has allowed bees to be kept in a greater range of environments while increasing the speed, rate and number of colonies (and pathogens) transported across diverse geographical distances. Currently, the transportation of honey bee colonies has reached a global scale, with thousands of colonies being moved across large distances [101]. The international movement of colonies for honey bee and bee product trade implies transportation of the bees and the viruses they carry. Human-mediated movements can explain the global distribution of most of the pathogens infecting the honey bees. For instance, international trade allowed the expansion of the ectoparasitic mite *V. destructor*, a biological or mechanical vector of honey bee viruses such as SBPV, ABPV and DWV [63]. Boosted by the direct transmission via the mite, and with a competitive advantage over other Varroa-transmitted viruses [102,103], DWV re-emerged to epidemic levels (Box 2) [83,104].

An example of large-scale colony transportation is the U.S. migratory beekeeping practice (Box 3). These massive movements and the high concentration of colonies could represent ideal conditions for the spread of honey bee viruses to a rate beyond their natural dissemination range. Besides migratory beekeeping, the international trade of queens and bee packages can also be accountable for the dissemination of bee viruses. Because the rules allow for the introduction of honey bees in cages containing one single queen with a maximum of 20 attendants, the transfer of viruses is unconstrained. Chen et al. [105] showed in a survey that honey bee queens may carry several viruses at the same time (up to five of six viruses screened). Veterinary authorities of many countries require the presentation of an international veterinary certificate attesting that imported honey bees come from apiaries located in a country or zone free from diseases. Until now, no viral diseases have been added to this list. However, each country may have its own list of forbidden or notifiable pathogens and parasites. For instance, in Europe, council directive 92/65/EEC of 13 July, 1992 specifies animal health requirements for bee trading (*Apis mellifera* and *Bombus* spp.), and all trades must be registered through TRACES (TRAde Control and Expert System, https://webgate.ec.europa.eu/sanco/traces/).

The dissemination of viruses is not limited to the trade of live bees. In the case of artificial insemination of queens, the international trade of semen from drones with desirable apicultural traits is becoming a common practice. As honey bee viruses have been reported in semen [106], in venereal infection of the queen [94,107] and via transmission to the offspring [108], this practice may impose a high risk for the transmission of viruses as it is virtually impossible to produce “virus-free” honey bee stocks. However, no data are currently available on the size and extent of this trade. Honey bee products have also been demonstrated to carry viruses and produce infection in exposed bees [109]; despite the exposure for several weeks of combs to the sun, DWV remains infectious in pollen and honey. More studies are needed on bee products and their storage to assess the contribution of hive products to the dissemination of viruses by the international trade.

Box 3 **The U.S. migratory beekeeping practice.**

Since the beginning of the 20th century, migratory beekeeping in the United States has blossomed to a massive agro-industry that is an essential component of U.S. agriculture. The pollination of almonds in California entails the largest migratory beekeeping event in the United States. Every year, leading up to the almond pollination season in the San Joaquin Valley of central California in February, about two-thirds of the 2.5 million commercially managed colonies, comprising approximately 30 billion honey bees are loaded onto flatbed trailers and trucked from various regions of the United States to participate in the single largest annual pollination event in America. This occasion leads to the production of millions of nuts, nearly 1 billion kilograms of biomass, which translates to upwards of 80% of the total almonds harvested worldwide in a single year (www.nass.usda.gov).

This mass migratory movement is both staggering and daunting as these bees must pollinate trillions of flowers within the valley in a small window of time to support the multibillion-dollar agro-economic industry in the region. While the start of the year marks the most lucrative pollination service provided by migratory commercial honey bees, it is merely the first stop on a continuous pollination journey that spans the four corners of the United States. By the end of fall, leading into winter, migratory commercial beekeepers invariably must find a locale that is temperate enough for their bee colonies to successfully survive the intervening months leading up to the next February almond event, when their annual journey of thousands of miles begins anew.

However, large pollination events bring bees and colonies from all over the country and with unknown pathogen loads into close contact: a perfect storm for epidemic disease transmission. One study showed that BQCV and SBV are significantly more prevalent in migratory bees [110], and this was confirmed for colonies being transported for almond pollination [111]. However, in the latter study, BQCV levels were similar between the migratory and the stationary colonies one month after they returned to their home apiaries. These results suggest that disease transmission may be greater for migratory colonies than for those in stationary apiaries.

### 3.2. Current Geographical Distribution

**Figure 1:**
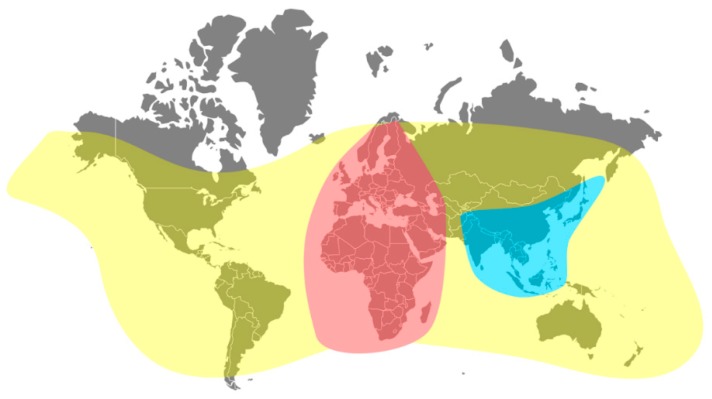
Distribution of domesticated honey bees (*A. mellifera* and *A. cerana*). Native and current distribution of the Western (*A. mellifera*) and Eastern (*A. cerana*) honey bees. Red: native range of *A. mellifera*. Yellow: current distribution of *A. mellifera*. Blue: native range of *A. cerana*. Current distribution was derived from ‘research grade’ observations on iNaturalist (www.inaturalist.org). Native range of *A. mellifera* and *A. cerana* from [116,117].

**Figure 2:**
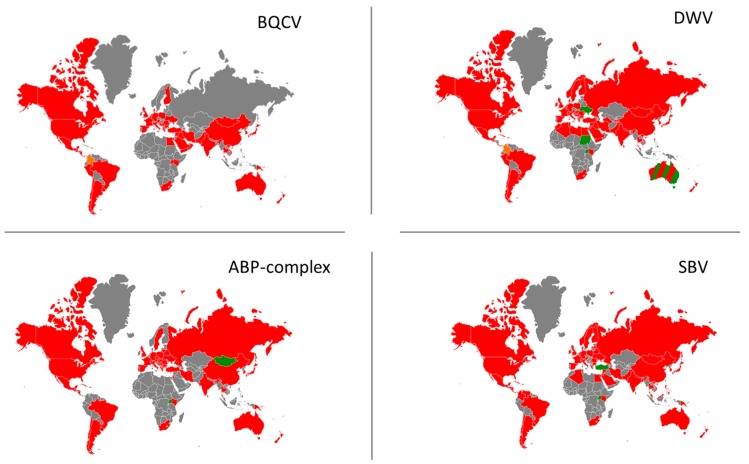
World maps depicting the viral distribution of DWV, BQCV, SBV and the acute bee paralysis complex in *A. mellifera*. Top left: global distribution of BQCV. Top right: global distribution of DWV. Bottom left: global distribution of the acute bee paralysis complex. Bottom right: global distribution of SBV. Red indicates that at least one study in the respective country has reported the presence of this virus. Green indicates that no study has detected the virus in their screening in the respective country. Orange indicates that the virus has been detected but only in bumblebees. Grey indicates absence of data.

Given the commercialization, transportation and resulting quasiglobal distribution of *A. mellifera* (Figure 1), many viruses of the honey bee can be found on multiple continents. In Figure 1, we show the native distribution of *A. mellifera* and *A. cerana*. Both honey bee species are currently the most used species in commercial apiculture [112,113]. In this review we focus on *A. mellifera*, although *A. cerana* and other *Apis* species are also potential hosts to the viruses reported here (reviewed in [114]). Summarizing the global distribution of a virus involves several limitations and difficulties, mostly due to the national and regional differences in how these are detected and reported. First, not all countries have the same means or techniques to screen for viruses or do not report their findings in readily accessible documents or formats, creating a knowledge gap for the presence or absence of the pathogens in different regions. For instance, there is a clear deficiency of studies that screen for viruses in African and Asian countries, even though beekeeping is present there. Secondly, reporting the absence of a certain virus in a location does not automatically render the whole country virus-free. Similarly, if a virus is detected, it does not mean that all the colonies in that country are infected. Finally, the honey bee host is not bound to national borders, nor to the precise localities where the viruses were reported, as hives may be relocated. For the purpose of this review, a general overview of the four most prevalent viruses or virus complexes is presented (Figure 2). Unsurprisingly, the most prevalent viruses are also those with a clear symptomatology, as these are more often reported and screened for. Although recently discovered viruses may well be less prevalent, these pathogens are also much less studied or screened for, creating a distorted view of their true distribution. One recent screening of apiaries in the USA showed however that two recently discovered viruses are widespread in the country [115]. Such screenings for new viruses should be encouraged for a comprehensive assessment of the global honey bee virome.

Our survey shows that DWV is the most investigated virus of *A. mellifera* (based upon the number of records in Web of Science), and is distributed almost globally. Only a few studies did not detect DWV, such as in the Ukraine [118], Sudan [119] and Kenya [61]. If these countries are truly free from this virus, keeping the DWV-free status will be nearly impossible as neighboring countries have reported its presence. Only truly remote and isolated territories, such as several island archipelagos [120] can be confidently considered devoid of DWV, as well as Australia, the largest DWV-free territory in the world (but see conflicting reports [63,109,121]). In pretty much all these cases, the absence of DWV is directly related to the strictly regulated and enforced absence of *V. destructor*. Another highly prevalent virus is SBV, which was not found in two investigated countries, Turkey [122] and Uganda [123]. Although BQCV is generally less studied than SBV, it was also distributed globally, and only a study in Syria [124] did not detect this virus. Viruses of the acute bee paralysis complex (i.e. ABPV, IAPV and KBV) also have a worldwide distribution [125], and only two studies, from Mongolia [126] and Uganda [123], screened for and did not detect these viruses.

### 3.3. Temporal Dynamics of Viruses Infecting Honey Bees

The dispersal and loads of different viruses are related to honey bee population dynamics and particularly their brood cycles. In temperate regions, colonies display dynamic annual cycles, where brood-rearing begins in mid-winter, reaches a peak in spring and ceases at the end of fall, dramatically impacting the in-hive demography and density. During this cycle, honey bee workers present a different physiology, activity and lifespan across the seasons. This annual cycle, driven by the succession of seasons and the availability of pollen and nectar, also has an impact on the life cycle of viruses (Table 1).

Early studies of honey bee pathology by Bailey et al. [37] revealed a peak prevalence during late spring and early summer for several common viruses, such as BQCV, ABPV, SBV and bee virus Y, as well as for other pathogens such as the gut microsporidia *Nosema apis*, followed by a general reduction in prevalence and abundance in fall and winter. More recent epidemiological studies have followed virus dynamics over time and confirmed this variation of virus prevalence across seasons [45,127,128,129,130]. Additionally, the U.S. National Honey Bee Disease Survey (2009–2014) has shown that annual virus prevalence peaks can shift from year to year. For example, peaks of IAPV prevalence were observed from early spring (February–April) to early summer (June–July) depending on the year [131]. Such temporal dynamics of incidence of infectious diseases are a common phenomenon, generally attributed to variations in environmental factors (e.g., temperature and daylight in temperate regions) and the subsequent changes in resource availability affecting host behavior, density, brood production and possibly host immune competence to fight pathogen infections [132,133,134,135,136].

The transmission route is another important factor that determines the dynamics of pathogens. Viruses can transmit in indirect ways, using vectors. The recent spread of *V. destructor* greatly modified the epidemiology of several viruses, which switched from low titers of mostly orally transmitted pathogens to rapidly spreading epidemics driven by the direct transmission and/or indirect effects of Varroa on individual and colony bee health [137]. Shortly after this invasion, viruses such as ABPV, DWV and SBPV were shown to be successfully transmitted by the mite [138]. Some of these viruses rapidly evolved towards a vector-borne lifestyle [78] with increased rates of prevalence and virulence [104,139]. As a result, the annual prevalence and titer patterns of these viruses were altered, with a peak later in the season, following the Varroa population dynamics within colonies [103,127,131,140,141,142]. Models show that transmission by the mite greatly modifies the in-hive dynamics of DWV, resulting in an imbalanced age structure of overwintering bees leading to the death of infected workers and eventually the colony during the following winter or spring [143]. With an early onset of the mite demographics, DWV loads and prevalence remain high at the start of the winter, which has a significant effect on bee and colony survival prior to the following spring and the start of a new brood cycle [75,144,145]. Given the influence of the mite population dynamics on the temporal variation of vectored viruses, any factor influencing the growth rate of the mite and its demographics will subsequently modify the dynamics of the viral population within the host. For instance, naturally mite-resistant colonies show a significant reduction of the mite population size relative to non-resistant colonies. Consequently, they display much lower DWV titers and different virus infection dynamics [51,146]. To minimize colony losses, the beekeeping industry has developed several strategies and tools to control mite populations infesting mite-susceptible honey bee stocks. Acaricide treatments have been shown to significantly reducte the prevalence of varroa-vectored viruses, including DWV and the ABPV-complex viruses [75,145]. Nevertheless, viruses are never totally eliminated from treated hives and can persist at low prevalence and titers, illustrating the importance of the multiple routes of transmission for disease dynamics.

Unfortunately, there are only a limited number of studies looking at the temporal variation of viruses in honey bee populations from tropical and subtropical regions. In these regions, honey bees and Varroa mites can reproduce all year round. Consequently, they would represent good controls for the effect of temporal variations on virus dynamics. Interestingly, at these latitudes, honey bees show remarkable resistance and tolerance to the mite, generally keeping mite infestation levels below the threshold required to kill colonies [147,148,149,150]. In their survey of diseases in honey bees of South Africa, Strauss et al. showed a temporal variation of BQCV, IAPV and DWV-B similar to that in temperate regions, but importantly observed no obvious signs of disease, suggesting that savannah honey bee populations may also be tolerant to these viruses [151].

Many viruses of honey bees are not vectored by ectoparasitic mites and remain exclusively orally transmitted (Table 1), even in mite-infested honey bee populations. Among them, some may not display a clear temporal pattern following the colony demographic development, such as CBPV. This virus is efficiently transmitted by close contact between infected and non-infected bees (e.g., crowding), and its development is therefore as much influenced by inclement weather or foraging opportunities as it is by host population development [152]. Additionally, some viruses may peak during winter months, as for LSV-2 and BVX [45], while others may not present any cyclic variation, including viruses with low prevalence [37].

Interestingly, virulence can determine the variations and population dynamics of vectored viruses. For example, the rapid death of workers induced by the systemic infection with ABPV is believed to result in a slower establishment of the disease within colonies relative to DWV, which displays a lower virulence under similar conditions [153]. Similarly, viral strains with higher virulence in adult workers may significantly shorten the lifespan of colonies and therefore express different temporal dynamics [64]. Recently, Remnant et al. [154] demonstrated that upon microinjection,, BQCV and SBV are so virulent for honey bee pupae that the host quickly dies, which may prevent their association with the ectoparasitic mites, since they are never transmitted in such a manner that the bee survives for this to be recorded. Ultimately, trade-offs may be reached between viruses, hosts and potential vectors to maximize fitness within their mutualistic interaction [95,155].

Finally, several viruses are shared among a large number of host species (see Section 2), and interspecies transmission of viruses may be frequent between co-foraging insect pollinators during spring and summer [156]. For these multihost pathogens, the temporal dynamics of infection in honey bees are more likely to depend on the life cycle of their main host species and the frequency of transmission to honey bees by host contact or shared food resources [137]. 

## 4. Discussion

*A. mellifera* is a species of significant ecological and economical relevance [157,158]. Consequently, this insect has been studied extensively in the past decades. Given the current threats honey bees face, a great share of this research has focused on stressors such as parasites and pathogens, aiming at conserving the host biodiversity and/or ameliorating the profitability of apiculture. In parallel, several features of the biology of *A. mellifera*, such as eusociality and social immunity, render this species a perfect study system to investigate fundamental epidemiological questions [159,160,161]. From the discovery of the first honey bee viruses in 1913 [162] and the pioneer studies by Bailey et al. [37], viruses have constantly intrigued bee scientists. The possibility to work with managed western honey bee colonies in controlled laboratory conditions or directly in the field makes it a very practical and popular study system. Thus, nowadays, over a century after the first reports of viruses infecting *A. mellifera* colonies, a multitude of studies has focused on these pathogens.

The quasiglobal prevalence of the major viruses of *A. mellifera* demonstrates the influence of the trade of honey bees and bee products across large scales. The distribution of these pathogens can also be facilitated by the introduction or invasion of vectors or alternative hosts to the pathogens [163,164,165]. The dispersal of viruses in novel environments is likely to result in the rapid spread of disease-causing agents in dense populations of *A. mellifera*. Honey bee colonies host tens of thousands of individuals that spend about half of their adult life as foragers, whose prime task is to collect resources on a variety of sites. During foraging flights, they are in contact with great diversity of plant and insect species outside the hives. Once infected, a forager returning to its colony provides a formidable means of transport for pathogens, which may then infect a new nest with plenty of resources to thrive. Thus, due to the anthropic movement of species at medium to large scale, and to the biology of their host at the small scale, viruses of honey bees are therefore extremely mobile.

**Figure 3:**
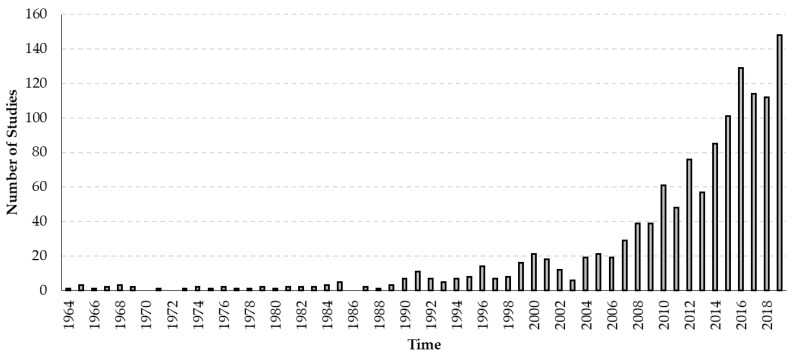
Increase of the number of studies on viruses of honey bees. Graph representing the temporal evolution of the number of studies published in scientific peer-reviewed journals.

With this work, one of our aims was to provide an extensive database (Table S1) reporting the global prevalence of viruses of the honey bees that can be used as a reference for future studies. The honey bee epidemiology research field is continuously expanding, as illustrated by the growing number of studies on honey bee viruses (Figure 3). Yet, several major knowledge gaps remain. For instance, data on the prevalence of viruses of honey bees are still lacking in several areas of the world. This absence of data is particularly remarkable in central Africa and some regions of Asia. Although we may have missed some reports in our survey as we only included peer-reviewed articles published in scientific journals, we can predict that the viruses found in the south and the north of the African continent may also be widespread in the center. In fact, the natural high swarming tendencies of native African *A. mellifera* subspecies may enforce the dispersal of diseases in that continent [166]. 

The continuous discovery of new viruses adds to the complexity of the honey bee pathosphere. Improving our understanding of the symptoms, virulence and epidemiology of these recently described agents is primordial to assess the potential threats they pose on their hosts. Furthermore, there is increasing evidence that viruses first described in honey bees are not restricted to this host and can be transmitted between genera [28,30,156]. Symptoms caused in well-studied bee species are wing deformities [167,168], reduced reproductive success [169] and mortality [156], but the impact on wild bee health is more difficult to assess, since most of these are solitary species that cannot be reared artificially. The commercialization of certain pollinators provides the opportunity for pathogens to spread both within managed honey bees or other commercial pollinators, as well as to wild pollinators. Therefore, the spillover of pathogens from imported pollinators to the natives bee species has to be taken in consideration as a threat of EIDs [137,170].

Additionally, the interactions between viruses of the honey bees, and between these agents and other biotic and abiotic stressors, are still poorly understood. For instance, new methods such as the metabolomics approach based on high-resolution mass spectrometry [171] may help in investigating how a host metabolism is altered during the course of virus infection in host cells and the changes characteristic for the transition from persistence to pathogenicity. 

Finally, knowledge on the transmission routes and on the biology of vectors and alternative host species will be needed to help prevent the spread and host switch of new viruses. The mechanisms of dispersal of viruses of honey bees could be investigated by studying the prevalence and biogeography of newly described viruses that do not yet occur globally. The spread of invasive species such as *Vespa velutina* and *Apis florea* [113,168] and vectors such as *V. destructor* and *T. mercedesae* is also alarming, as these species are known to be infected by viruses of *A. mellifera* [138,172,173].

## 5. Conclusions

The study of viruses of *A. mellifera* remains a key area for the conservation of this economically and ecologically important pollinator, along with the many other species the western honey bee cohabits with across the globe. The knowledge on the diversity and distribution of these key pathogens will contribute to our understanding of these important organisms and help to prevent the spread of additional emerging infectious diseases (EIDs). The database provided and the knowledge gaps highlighted in this review will help designing new projects to study the biology and dissemination of viruses of honey bees across their multiple hosts.

## Figures and Tables

**Table 1:** Viruses detected in *A. mellifera*. List of all viruses identified thus far in *A. mellifera* together with their (provisional) name, acronym, taxonomic classification and genome accession number (preference for earliest reported full-length genome sequence; n.a. = “not available”; *PEHZ00000000* is the accession number of the entire raw sequence data of Galbraith et al. [61] containing these virus sequences). Distinct taxonomic units (Order, Family, Virus, Major Strain) are separated by horizontal lines. Viruses with partial genome sequences are marked with a single asterisk (*), and those for which no sequence information is available are marked with two asterisks (**). For all other viruses (near) full-length genome sequences are available. ^a^: Viruses detected primarily in *V. destructor*. ^b^: Viruses with DNA genomes. ^c^: Novel picorna-like viruses identified in Australian honey bees by Roberts et al. [50]. The columns on the right summarize the current experimental evidence for the presence (+, highlighted), unclear (~), absence (-) or unknown (?) status of the different transmission routes for the various viruses (green = oral-fecal transmission; red = varroa-mediated transmission; black = contact transmission; blue = sexual-vertical transmission; orange = environmental transmission), as well as the evidence for their seasonal incidence (spring, summer, autumn, winter), marked on a three-point scale (+, ++, +++).

Genome	Order	Family	Virus	Major Strains	Accession Number	TRANSMISSION					SEASON				
						ORAL-FECAL	VARROA	CONTACT	VERTICAL	ENVIRONMENT		SPRING	SUMMER	AUTUMN	WINTER
ssRNA(+)	Picornavirales	Dicistroviridae	Acute bee paralysis virus	ABPV	AF150629	**+**	**+**	**-**	**+**	**+**		**+**	**+++**	**++**	**+**
				KBV	AY275710	**+**	**+**	**-**	**+**	**+**		**+**	**++**	**+++**	**?**
				IAPV	EF219380	**+**	**+**	**-**	**+**	**+**		**+**	**++**	**++**	**?**
			Aphid lethal paralysis virus	ALPV	AF536531	**?**	**?**	**?**	**?**	**+**		**-**	**+++**	**-**	**?**
			Apis dicistrovirus	ADV	KY354239	**?**	**?**	**?**	**?**	**?**		**?**	**?**	**?**	**?**
			Big Sioux river virus	BSRV	KY826434	**?**	**?**	**?**	**?**	**+**		**-**	**+++**	**++**	**?**
			Black queen cell virus	BQCV	AF183905	**+**	**~**	**-**	**+**	**+**		**+**	**+++**	**+**	**+**
			Bundaberg bee virus 1 ^c^	QLD-6	MG995706	**?**	**?**	**?**	**?**	**?**		**?**	**?**	**?**	**?**
			Bundaberg bee virus 2 * ^c^	QLD-4	MG995700	**?**	**?**	**?**	**?**	**?**		**?**	**?**	**?**	**?**
			Empeyrat virus ^c^	NT-5	KU754505	**?**	**?**	**?**	**?**	**?**		**?**	**?**	**?**	**?**
				QLD-7	MG995702	**?**	**?**	**?**	**?**	**?**		**?**	**?**	**?**	**?**
			Hobart bee virus 1 * ^c^	TAS-7	MG995722	**?**	**?**	**?**	**?**	**?**		**?**	**?**	**?**	**?**
			Perth bee virus 1 * ^c^	WA2-13	MG995730	**?**	**?**	**?**	**?**	**?**		**?**	**?**	**?**	**?**
			Perth bee virus 2 * ^c^	WA1-14	MG995726	**?**	**?**	**?**	**?**	**?**		**?**	**?**	**?**	**?**
			Renmark bee virus 1 * ^c^	SA-7	MG995710	**?**	**?**	**?**	**?**	**?**		**?**	**?**	**?**	**?**
			Rhopalosiphum padi virus ^c^	RhPV	AF022937	**?**	**?**	**?**	**?**	**?**		**?**	**?**	**?**	**?**
			Robinvale bee virus 1 * ^c^	VN1-10	MG995714	**?**	**?**	**?**	**?**	**?**		**?**	**?**	**?**	**?**
			Robinvale bee virus 2 * ^c^	VN1-57	MG995719	**?**	**?**	**?**	**?**	**?**		**?**	**?**	**?**	**?**
			Robinvale bee virus 3 * ^c^	VN1-50	MG995718	**?**	**?**	**?**	**?**	**?**		**?**	**?**	**?**	**?**
		Iflaviridae	Bundaberg bee virus 4 ^c^	QLD-11	MG995705	**?**	**?**	**?**	**?**	**?**		**?**	**?**	**?**	**?**
			Bundaberg bee virus 5 ^c^	QLD-13	MG995706	**?**	**?**	**?**	**?**	**?**		**?**	**?**	**?**	**?**
			Bundaberg bee virus 6 ^c^	QLD-14	MG995707	**?**	**?**	**?**	**?**	**?**		**?**	**?**	**?**	**?**
				NT-12	MG995697	**?**	**?**	**?**	**?**	**?**		**?**	**?**	**?**	**?**
			Darwin bee virus 2 * ^c^	NT-6	MG995694	**?**	**?**	**?**	**?**	**?**		**?**	**?**	**?**	**?**
				NT-17	MG995699	**?**	**?**	**?**	**?**	**?**		**?**	**?**	**?**	**?**
			Deformed wing virus	DWV-A	AY292384	**+**	**+**	**-**	**+**	**+**		**+**	**++**	**+++**	**+++**
				DWV-B	AY251269	**+**	**+**	**-**	**+**	**+**		**+**	**+**	**++**	**+**
				DWV-C	ERS657948	**?**	**?**	**?**	**?**	**?**		**+**	**+**	**+++**	**+++**
			(Egypt bee virus*)	DWV-D	n.a.	**?**	**?**	**?**	**?**	**?**		**?**	**?**	**?**	**?**
			Moku virus	MV	KU645789	**?**	**?**	**?**	**?**	**?**		**?**	**?**	**?**	**?**
			Perth bee virus 3 * ^c^	WA2-20	MG995731	**?**	**?**	**?**	**?**	**?**		**?**	**?**	**?**	**?**
				VN2-2	MG995723	**?**	**?**	**?**	**?**	**?**		**?**	**?**	**?**	**?**
				VN2-6	MG995724	**?**	**?**	**?**	**?**	**?**		**?**	**?**	**?**	**?**
			Renmark bee virus 2 * ^c^	SA-5	MG995709	**?**	**?**	**?**	**?**	**?**		**?**	**?**	**?**	**?**
			Robinvale bee virus 4 ^c^	VN3-43	MG995721	**?**	**?**	**?**	**?**	**?**		**?**	**?**	**?**	**?**
			Sacbrood virus	SBV	AF092924	**+**	**-**	**-**	**?**	**+**		**+++**	**++**	**+**	**?**
				TSBV	KM884995	**+**	**?**	**?**	**?**	**?**		**?**	**?**	**?**	**?**
			Slow bee paralysis virus	SBPV	EU035616	**+**	**+**	**-**	**?**	**+**		**-**	**-**	**-**	**?**
			*Varroa destructor* virus-2 ^a^	VDV-2	KX578271	**?**	**?**	**?**	**?**	**?**		**?**	**?**	**?**	**?**
		Nodaviridae	Apis Nora virus	ANV	KY354240	**?**	**?**	**?**	**?**	**?**		**?**	**?**	**?**	**?**
			Nodamura-like virus *	?	*PEHZ00000000*	**?**	**?**	**?**	**?**	**?**		**?**	**?**	**?**	**?**
		Secoviridae	Seco-like virus *	?	*PEHZ00000000*	**?**	**?**	**?**	**?**	**?**		**?**	**?**	**?**	**?**
			Tobacco ringspot virus	TRSV	U50869	**?**	**?**	**?**	**?**	**?**		**?**	**?**	**?**	**?**
		?	Arkansas bee virus **	ArkBV	n.a.	**?**	**?**	**?**	**?**	**?**		**?**	**?**	**?**	**?**
			Berkeley bee virus **	BerkBPV	n.a.	**?**	**?**	**?**	**?**	**?**		**?**	**?**	**?**	**?**
		?	Bundaberg bee virus 7 * ^c^	QLD-8	MG995703	**?**	**?**	**?**	**?**	**?**		**?**	**?**	**?**	**?**
			Darwin bee virus 5 ^c^	NT1	MG995692	**?**	**?**	**?**	**?**	**?**		**?**	**?**	**?**	**?**
			Perth bee virus 6 * ^c^	WA2-62	MG995732	**?**	**?**	**?**	**?**	**?**		**?**	**?**	**?**	**?**
			Perth bee virus 7 * ^c^	WA1-16	MG995727	**?**	**?**	**?**	**?**	**?**		**?**	**?**	**?**	**?**
			Perth bee virus 8 * ^c^	WA1-18	MG995728	**?**	**?**	**?**	**?**	**?**		**?**	**?**	**?**	**?**
			Perth bee virus 9 * ^c^	WA1-9	MG995725	**?**	**?**	**?**	**?**	**?**		**?**	**?**	**?**	**?**
			Renmark bee virus 4 ^c^	SA-10	MG995708	**?**	**?**	**?**	**?**	**?**		**?**	**?**	**?**	**?**
			Renmark bee virus 5 * ^c^	SA-8	MG995711	**?**	**?**	**?**	**?**	**?**		**?**	**?**	**?**	**?**
			Robinvale bee virus 7 * ^c^	VN1-35	MG995717	**?**	**?**	**?**	**?**	**?**		**?**	**?**	**?**	**?**
			Robinvale bee virus 9 * ^c^	VN3-31	MG995720	**?**	**?**	**?**	**?**	**?**		**?**	**?**	**?**	**?**
		?	Darwin bee virus 6 ^c^	NT-8	MG995696	**?**	**?**	**?**	**?**	**?**		**?**	**?**	**?**	**?**
			Perth bee virus 4 * ^c^	WA2-63	MG995733	**?**	**?**	**?**	**?**	**?**		**?**	**?**	**?**	**?**
			Perth bee virus 5 * ^c^	WA1-24	MG995729	**?**	**?**	**?**	**?**	**?**		**?**	**?**	**?**	**?**
			Renmark bee virus 3 ^c^	SA4	MG995708	**?**	**?**	**?**	**?**	**?**		**?**	**?**	**?**	**?**
			Robinvale bee virus 5 * ^c^	VN1-15	MG995715	**?**	**?**	**?**	**?**	**?**		**?**	**?**	**?**	**?**
			Robinvale bee virus 8 ^c^	VN1-22	MG995716	**?**	**?**	**?**	**?**	**?**		**?**	**?**	**?**	**?**
		?	Bundaberg bee virus 8 * ^c^	QLD-9	MG995704	**?**	**?**	**?**	**?**	**?**		**?**	**?**	**?**	**?**
			Darwin bee virus 7 * ^c^	NT-15	MG995698	**?**	**?**	**?**	**?**	**?**		**?**	**?**	**?**	**?**
		?	Darwin bee virus 8 * ^c^	NT-7	MG995695	**?**	**?**	**?**	**?**	**?**		**?**	**?**	**?**	**?**
			Robinvale bee virus 6 * ^c^	VN1-8	MG995713	**?**	**?**	**?**	**?**	**?**		**?**	**?**	**?**	**?**
	Tymovirales	Tymoviridae	Bee Macula-like virus	BeeMLV	KT162925	**?**	**+**	**?**	**?**	**+**		**+**	**++**	**+++**	**?**
			Varroa Tymo-like virus * ^a^	VTLV	KT162926	**?**	**+**	**?**	**?**	**?**		**?**	**?**	**?**	**?**
	?	?	Cloudy wing virus **	CWV	n.a.	**?**	**-**	**~**	**?**	**?**		**+**	**+**	**+**	**?**
	?	?	Chronic bee paralysis virus	CBPV	EU122230	**+**	**-**	**+**	**?**	**+**		**++**	**++**	**+**	**?**
		?	Bee virus **	BVX	n.a.	**+**	**?**	**?**	**?**	**?**		**+++**	**+**	**+**	**?**
				BVY	n.a.	**+**	**?**	**?**	**?**	**?**		**+**	**+++**	**+**	**?**
			Lake Sinai virus	LSV-1	HQ871931	**?**	**?**	**?**	**?**	**+**		**++**	**+++**	**++**	**?**
				LSV-2	HQ888865	**?**	**?**	**?**	**?**	**+**		**+++**	**+**	**+**	**?**
				LSV-3	MH267700	**?**	**?**	**?**	**?**	**+**		**+**	**+**	**+**	**?**
				LSV-4	KM886903	**?**	**?**	**?**	**?**	**+**		**+**	**+**	**+**	**?**
	?	?	*Varroa destructor* virus-3 ^a^	VDV-3	KX578272	**?**	**?**	**?**	**?**	**?**		**?**	**?**	**?**	**?**
		?	*Varroa destructor* virus-4 ^a^	VDV-4	MK032464	**?**	**?**	**?**	**?**	**?**		**?**	**?**	**?**	**?**
ssRNA(-)	Articulavirales	Orthomyxoviridae	Varroa orthomyxovirus-1 ^a^	VOV-1	MK032465	**?**	**?**	**?**	**?**	**?**		**?**	**?**	**?**	**?**
	Bunyavirales	Arenaviridae	Apis bunyavirus-1 *	ABV-1	KY354236	**?**	**?**	**?**	**?**	**?**		**?**	**?**	**?**	**?**
		Phasmaviridae	Apis bunyavirus-2 *	ABV-2	KY354237	**?**	**?**	**?**	**?**	**?**		**?**	**?**	**?**	**?**
	Mononegavirales	Rhabdoviridae	Apis rhabdovirus-1	ARV-1	MH267691	**?**	**?**	**?**	**?**	**?**		**?**	**?**	**?**	**?**
			Apis rhabdovirus-2	ARV-2	KY354234	**?**	**?**	**?**	**?**	**?**		**?**	**?**	**?**	**?**
dsRNA	?	Partitiviridae	Partiti-like virus *	?	*PEHZ00000000*	**?**	**?**	**?**	**?**	**?**		**?**	**?**	**?**	**?**
ssDNA	?	Circoviridae	Circo-like virus-1 * ^b^	?	*PEHZ00000000*	**?**	**?**	**?**	**?**	**?**		**?**	**?**	**?**	**?**
			Circo-like virus-2 * ^b^	?	*PEHZ00000000*	**?**	**?**	**?**	**?**	**?**		**?**	**?**	**?**	**?**
dsDNA	Megavirales	Baculoviridae	*Apis mellifera* filamentous virus ^b^	AmFV	MH243376	**+**	**?**	**?**	**?**	**?**		**+++**	**+**	**+**	**?**
		Iridoviridae	Apis iridescent virus * ^b^	AIV	AF042340	**?**	**?**	**?**	**?**	**?**		**+**	**++**	**+**	**?**

**Table 2:** Symptoms of viral infections in *A. mellifera*. Tissue tropism refers to the organs in which the virus was found. Symptoms report the physical and physiological effect of viruses as observed in honey bees. Only viruses with known symptoms are reported here.

Virus	Tropism	Symptoms	Refs
*Acute bee paralysis virus* complex	Nervous system, cytoplasm of fat body cells, brain and hypopharyngeal glands	Trembling, inability to fly, gradual darkening and loss of hair from the thorax and abdomen, crawling on the ground and upward on grass, rapid death for highly infected bees	[84,85,86]
*Apis iridovirus*	*NA*	Iridescence of most internal organs	[87]
*Apis mellifera filamentous virus*	*NA*	Milky-white hemolymph	[88]
*Bee virus X*	*NA*	Shortened lifespan of adult bees	[89]
*Bee virus Y*	*NA*	Shortened lifespan of adult bees	[37]
*Black queen cell virus*	Gut tissue	Yellowish queen larvae with sac-appearance that resembles SBV and with time evolves to dark brown, infected pupae turn brown and die, dark brown to black colored walls in queen cells, significantly shortened life span in adult bees	[36,84,90]
*Chronic bee paralysis virus*	Nervous system, alimentary tract, mandibular and hypopharyngeal glands	Syndrome 1: trembling of the wings and bodies, bloated abdomen, inability to fly, crawling on the ground and upward on grass, gather in groups in the warmest areas of the nest, death within few days	[84,91]
		Syndrome 2 (’black robbers’): hairless (thus appearing smaller), darker, greasy in appearance, shiny, suffer nibbling attacks by the healthy bees, death within few days	
*Cloudy wing virus*	Tracheal tissue and thoracic muscles	Opaque wings, shortened lifespan of adult bees	[92,93]
*Deformed wing virus*	Whole body, including the queen ovaries, queen fat body, spermatheca, and drone seminal vesicles, tissues of wings, head, thorax, legs, hemolymph and gut	Crumpled or aborted wings, shortened abdomens, paralysis, severely shortened adult life span for emerging worker and drone bees, modified responsiveness to sucrose, impaired learning, impaired foraging behavior	[59,64,94,95,96]
*Invertebrate iridescent virus* Type 6	*NA*	Flightless clustering bees	[87]
*Sacbrood virus*	Hypopharyngeal glands of worker bees, cytoplasm of fat, muscle and tracheal-end cells of larvae	Pupation failure, ’sac’ phenotype: swollen larvae filled with ecdysial fluid full of viral particles, precocious foraging, reduction of adult life span and metabolic activities, impaired foraging activity	[97,98,99]
*Slow bee paralysis virus*	Nervous system	Paralysis of the two anterior legs a day or two before death	[89]

## Supplementary Materials

The following are available online at https://www.mdpi.com/2075-4450/11/4/239/s1, Table S1: Distribution of viruses of *Apis mellifera*.
